# Pharmacological and immunological effects of praziquantel against *Schistosoma japonicum*: a scoping review of experimental studies

**DOI:** 10.1186/s40249-018-0391-x

**Published:** 2018-02-07

**Authors:** Shu-Hua Xiao, Jun Sun, Ming-Gang Chen

**Affiliations:** 1 National Institute of Parasitic Diseases, Chinese Center for Disease Control and Prevention, Key Laboratory of Parasite and Vector Biology, Ministry of Health, WHO Collaborating Centre for Tropical Diseases, Shanghai, 200025 People’s Republic of China; 2 Institute for Infectious Disease and Vaccine Development, Tongji University School of Medicine, Shanghai, 200092 People’s Republic of China

**Keywords:** Praziquantel, Pharmacological action, Immunological action, *Schistosoma japonicum*, Experimental study, Review

## Abstract

**Background:**

Chemotherapy for schistosomiasis has been around for 100 years. During the past century, great efforts have been made to develop new antischistosomal drugs from antimonials to nonantimonials, and some of these have been used extensively in clinical treatment. With the exception of a few drugs, such as oxamniquine and metrifonate, most of the antischistosomals developed in the pre-praziquantel period have variable limitations with respect to safety and efficacy. Although oxamniquine and metrifonate have been used for schistosomiasis control, they are only effective against *Schistosoma mansoni* and *S. haematobium*, respectively. Currently, praziquantel is the only drug used for treatment of all five species of human schistosomes. In this review, the pharmacological and immunological effects of praziquantel against *S. japonicum* are summarized and discussed.

**Main text:**

From the end of the 1970s until the 2000s, scientists have conducted a series of experimental studies on the effects of praziquantel against *S. japonicum*. These have included examining its unique pharmacological action on schistosomes, the characteristics in susceptibility of the different developmental stages of schistosomes to the drug, the relationship between plasma concentration of the drug and efficacy, the impact of host factors on cidal action of the drug, prevention and early treatment of schistosomal infection, as well as praziquantel-resistant schistosomiasis.

**Conclusion:**

The effects of praziquantel against *S. japonicum*, as elucidated by the experimental studies that are reviewed in this paper, may have some reference significance for the development of new antischistosomals.

**Electronic supplementary material:**

The online version of this article (10.1186/s40249-018-0391-x) contains supplementary material, which is available to authorized users.

## Multilingual abstacts

Please see Additional file 1 for translations of the abstract into the five official working languages of the United Nations.

## Background

Five species of schistosomes, i.e., *Schistosoma japonicum, S. haematobium, S. mansoni, S. intercalatum*, and *S. mekongi*, are the major species that can infect humans. Despite scientists recognizing the symptoms of schistosomiasis earlier than the discovery of its pathogen [1], real chemotherapy was undertaken at a much later stage.

Globally, the era of chemotherapy to treat schistosomiasis began when a human case with *S. haematobium* infection was successfully treated in 1918 with potassium antimony tartrate (PAT) [2]. Since then, many different categories of antischistosomal drugs have been developed, and some of them, including sodium antimony subgallate (Sb-273), lucanthone, hycanthone, amoscanate, metrifonate, oxamniquine, niridazole, furapromidum, and hexachloroparaxylene have been used extensively in the treatment of schistosomiasis [3–8]. With the exception of a few drugs, such as oxamniquine and metrifonate, most of the antischistosomals developed in the pre-praziquantel period have variable limitations with respect to safety and efficacy. Moreover, oxamniquine and metrifonate are only effective against *S. mansoni* and *S. haematobium*, respectively.

Since the discovery of praziquantel at the end of the 1970s, great progress in schistosomiasis control has been achieved worldwide. This is due to its good levels of tolerance and safety, excellent acceptability by patients, easy administration at a single-dose or very short treatment course (1–2 days), good efficacy profile against all schistosomes parasitizing humans [4–6, 9–11], and low cost [5, 12].

Currently, schistosomiasis is distributed in 78 countries in Africa, Asia, and South America. It is estimated that 779 million people live in endemic areas and about 280 million people are infected with schistosomes [13]. In 2006, the strategy for schistosomiasis control proposed by the World Health Organization was to reduce disease burden, i.e., periodic treatment of at-risk populations with praziquantel (preventive chemotherapy), which aimed to cure mild symptoms and prevent infected people from developing severe, late-stage chronic disease [14–16]. Even though re-infection may occur after treatment, as praziquantel is only effective against the very early stage of juvenile (three-hour old worm, day 0 schistosomula) and adult worms but has little effect against young developing stages of the parasites [17–19], the risk of developing severe disease is diminished and even reversed when treatment is started early in life [14].

Since the end of the 1970s until the 2000s, scientists have conducted a series of experimental studies on the effects of praziquantel against *S. japonicum*. These have included examining its unique pharmacological action on schistosomes, the characteristics in susceptibility of different developmental stages of schistosomes to the drug, the relationship between plasma concentration of the drug and efficacy, the impact of host factors on cidal action of the drug, prevention and early treatment of schistosomiasis, as well as praziquantel-resistant schistosomiasis. In this review, relevant data on these studies are summarized.

## Unique pharmacological effects of praziquantel against schistosomes

Praziquantel has three unique pharmacological effects on *S. japonicum* and *S. mansoni,* i.e., stimulation of worm motor activity, spasmodic contraction of the musculature, and vesicle formation in tegument [20–25]. The former two actions link the subsequent hepatic shift of the worms in vivo, while the latter is key to kill the worm.

### Stimulation of worm motor activity and spasmodic contraction of musculature

The minimal effective concentrations (MECs) of praziquantel required to induce increased motor activity followed by contraction of various developmental stages of *S. mansoni* and *S. japonicum* in vitro are similar, i.e., 0.005–0.1 μg/ml and 0.005–0.05 μg/ml, respectively (see Table 1). With these MECs of praziquantel, the increased activity of parasites occurs almost immediately and the contraction of the worm musculature typically begins after a short lag phase. At a higher concentration of 1 or 10 μg/ml, marked contraction and paralysis masks the early stimulation phase [25, 26]. Praziquantel-induced stimulation of motor activity of adult *S. japonicum* worms is similar to the effect of serotonin (5-HT) on the worms in many aspects. However, praziquantel neither increases the endogenous 5-HT of schistosomes nor enhances uptake of exogenous 5-HT of the worm. Praziquantel likely displays 5-HT-like action or is a 5-HT receptor agonist [23, 27].

**Table 1:** The minimal effective concentrations (MECs) of praziquantel needed to induce an increased in motor activity, contraction of musculature or vesicle formation in different developmental stages of *S. mansoni* and *S. japonicum*

Stage	MEC (μg/ml) producing			Time of onset of vesiculation	Vesicle number^b^
	Stimulation	Contraction	Vesiculation^a^		
*S. mansoni*	Reference [26]				
Day 0	0.005	0.010	0.1	2–4 h	Few
Day 3	0.010	0.010	1.0	> 4 h	Few
Day 7	0.010	0.010	1.0	> 4 h	Few
Day 14	0.010	0.010	0.1	30 min	Many
Day 28	0.005	0.010	0.1	30 min	Many
Day 35	0.005	0.010	0.1	5–15 min	Very many
Day 42	0.005	0.010	0.1	5–15 min	Very many
*S. japonicum*	Reference [41]				
Day 0	0.01	0.05	0.5	3–4 h	Few
Day 3	0.01	0.05	1.0	24 h	Very few
Day 7	0.01	0.05	1.0	24 h	Very few
Day 14	0.01	0.05	1.0	2 h	Many
Day 21	0.01	0.05	0.1	2 h	Many
Day 28	0.005	0.05	0.1	2 h	Many
Day 35	0.005	0.05	0.1	15 min	Very many
Day 42	0.005	0.05	0.1	15 min	Very many

^a^Observed by light microscopy

^b^Few ≤ 5 vesicle per parasite, many = 5–20 vesicles per parasite, and very many ≥20 vesicles per parasite

Based on in vitro analyses using some neurotransmitters or various compounds known to interact with the schistosome’s neuroreceptive sites and relevant blocking agents, it is suggested that the spasmodic contraction of *S. mansoni* and *S. japonicum* induced by praziquantel does not happen through neurotransmitters [20, 24, 28]. Nevertheless, praziquantel causes a rapid rise in the tension of the musculature of male *S. mansoni* worms, which is related to the muscle cell membrane potential. The resting membrane potential (RMP) of a male worm’s muscle cell is − 30.7 ± 1.2 mV, but in the depolarization induced rapidly by praziquantel the RMP rises to 15.6 ± 3.1 mV. Since in the incubation medium without sodium ions (Na^+^), low concentration of calcium ion(Ca^2+^), or high concentration of magnesium ions (Mg^2+^), the contractile activity of worm musculature caused by praziquantel is blocked, the action of the drug on RMP of the worm’s muscle cells might be related to an increase in the tension of the parasite’s musculature [29, 30].

Microelectrode studies have indicated that the membrane potentials of male schistosomes are derived from these sources i.e., tegument membrane, muscle masses, and the basal lamina, interstitial fibers, and extracellular space surrounding the muscle. Further studies found that the rise of tegumental membrane RMP and muscle RMP caused by praziquantel was slow. Hence, it seems that praziquantel-induced contraction of schistosomes is not dependent on changes in the membrane potential [31, 32].

According to in vivo studies, these two pharmacological activities are related to the hepatic shift of schistosomes induced by praziquantel [25, 33]. The hepatic shift of schistosomes caused by praziquantel in vivo is so fast that 5 min after *S. japonicum*-infected mice are orally administered with praziquantel at a single curative dose of 300 mg/kg, 94.6% of the worms have been found to shift to the liver [34]. Meanwhile, other antischistosomal drugs, such as PAT, furapromidum, artemether, oxamniquine, mefloquine, and various ozonides such as OZ78, and OZ418 (synthetic 1,2,4-trioxolanes, secondary ozonides, or OZs) showed slower action in *S. japonicum* or *S. mansoni* infections with respect to hepatic shift or tegumental damage [35–40].

### Vesicle formation in the tegument of *Schistosoma* worms

Vesicle formation in the tegument of *Schistosoma* worms is one of the earliest and most important effects of praziquantel. It is probably correlated to subsequently killing the parasites and is also consistent with susceptibilities of various developmental stages of schistosomes to the drug. It has been shown through light microscopy (LM) that the immature developmental stages (3-, 7-, and 14-day-old *S. mansoni* and *S. japonicum* worms) are particularly resistant (see Table 1) [26, 41]. Scanning electron microscopy (SEM) has been used to observe tegument of various stages of schistosomula exposed to praziquantel and revealed that a concentration of 30 μg/ml insulted the tegument of day 0 schistosomula (skin stage) where swelling, fusion, and vesicle formation were recorded. However, day 3, day 7, and day 14 juvenile worms exhibited no or only slight tegument damage. Apparent damage to the tegument of day 21 schistosomula and day 28, day 35, and day 42 adult worms was seen 15 min after exposure to the same concentration of praziquantel that resulted in severe damage to the tegument, as was revealed 4–24 h later [19, 42]. All three aforementioned praziquantel-induced pharmacological effects have also been described for *S. mansoni* worms [43].

### Praziquantel-induced damage of developmental stages of *S. japonicum* worms and host cellular response

Examination of day 0, day 21, day 28, day 35, and day 42 schistosomes 0.5–24 h after oral administration of praziquantel (400 mg/kg) has revealed extensive vesiculation and rupture of the tegument, while little or only mild and local vesiculation was seen in the tegument of day 3, day 7, day 11, and day 14 schistosomula. Moreover, strong host cell response was found around damaged day 0 and adult schistosomes (see Table 2) [44]. Meanwhile, after praziquantel administration, alkaline phosphatase (AKP) activity and glycogen content in day 0 schistosomula has been shown to decrease significantly or even disappear, while in day 3 and day 7 schistosomula only a few showed a mild change in both AKP activity and glycogen content [45]. Four hours after administration of praziquantel using the same dose and route, observation using SEM revealed no apparent tegumental damage in day 3, day 7, and day 14 schistosomula, while slight or moderate tegument damage was detected in day 21 worms. On the contrary, severe tegument damage was observed in adult schistosomes [19, 42].

**Table 2:** Praziquantel-induced damage on tegument in different developmental stages of *S. japonicum*, harbouring in mice, and treated orally with the drug at a single dose of 400 mg/kg for 0.5–24 hours^a^

Developmental stages of schistosomes (d)	Percentages of worm tegument showing damage^b^		Host cell response expressed by dominant leukocytes^c^	
	Vesiculation	Rupture	Neutrophils	Lymphocytes
d0	41.8	49.2	47.5 ± 8.2	
d21, d28, d35, d42	20.4–38.7	17.9–56.2	26.8 ± 5.2–58.4 ± 12.2^d^	
d3, d11, d14	Normal tegument in most worms, or emergence of very few vesicles			12.8 ± 5.9–16.8 ± 5.0
d7	Ditto			2.5 ± 0.7

^a^Data from reference [44]

^b^2–3 mice harbouring different developmental stages of schistosomes killed 0.5, 1, 4 and 24 hr after treatment, skins (d0), lungs (d3) or livers (d11, d14, d21, d28, d35, d42) removed for routine preparation of tissue section

^c^Each value represents 40–80 sections

^d^The cells around the d21 schistosomules mainly lymphocytes

The results suggest that the lower efficacy of praziquantel in day 21 schistosomula might be attributed to the low level of antischistosome antibody present in the host 3 weeks after infection. Further observation of mice infected with cercariae for 21 days and treated orally with praziquantel at a higher daily dose of 500 mg/kg for 1–3 days revealed worm reduction rates of 43.8–90.4% [46]. Examination using SEM showed that under treatment with the higher daily dose of praziquantel for 3 days, day 21 schistosomula had severe swelling, erosion, and peeling of the tegument, accompanied by the attachment of the host leukocytes on the worm’s surface. This demonstrates that, if given at a higher daily dose for 3 days, praziquantel can directly kill day 21 schistosomula [47].

### Recovery of different developmental stages of schistosomes after exposure to praziquantel

Examination using LM has indicated that *S. japonicum* at different developmental stages exposed to a praziquantel dose of 30 μg/ml for 15 min and then transferred to a drug-free medium for another 24 h results in a spasmodic contracted worm body that was elongated with normal motor activity on day 0, day 3, day 7, day 14, day 21, and day 28. In day 35 and day 42 adult worms, relaxation of contracted musculature was also seen, but numerous vesicles were still observed in the tegument. If the exposure period was extended to 4 h, day 3, day 7, day 14, and day 21 schistosomula recovered completely, while day 35, day 42, and some day 0 and day 28 worms still revealed abnormalities [41]. Similar results were obtained by SEM examination, except that a few day 21 schistosomula had severe tegumental injury [42]. Stage-specific difference also occurred in *S. mansoni* worms [26].

### Stage-specific susceptibility of *S. japonicum* worms to praziquantel and exposure of worm surface antigen

Praziquantel has no apparent therapeutic effect in mice infected with day 3–day 21 *S. japonicum* schistosomula, but it does have an effect on day 0 schistosomula and day 28–day 42 adult schistosomes [17, 19]. Observation using transmission electron microscopy (TEM) and SEM has indicated that 5–30 min after exposure of adult *S. japonicum* worms to praziquantel in vitro or in vivo, the tegument of the worm showed extensive damage, including emergence of numerous ball-like structures or bulbs in the ridges, swelling and rupture of cytoplasmic processes, destruction of the nuclei of the syncytium, decrease in tegumental AKP, and erosion and peeling of the tegumental surface, followed by the attachment of host leukocytes to the denuded surface [48–52].

It has been demonstrated that adult schistosomes may acquire the molecules of host origin (host antigens) that incorporated into their surface, which results in the evasion of host immune reaction [53]. Since praziquantel is able to quickly damage the tegument of schistosomes, exposure of worm surface antigen will follow. Ten to 30 min after administration of praziquantel at a dose of 300 mg/kg given to adult *S. japonicum*-infected mice and using the indirect fluorescent antibody technique (IFAT), exposure of worm surface antigen extended gradually to 1/3–1/2 of the worm’s surface in 6 h. The speed, degree, and extent of antigen exposure in schistosomes coincides with the tegument alteration, as observed by SEM, and these are dose-dependent [23, 50, 54]. Further examination using the IFAT indicated that 4 h after oral administration of praziquantel at a dose of 400 mg/kg given to mice infected with various developmental stages of *S. japonicum*, the percentage of exposure of worm surface antigen in day 0, day 3–day 14, day 21, day 28, and day 42 were 89.8%, 0–10%, 32.9%, 25.7%, and 41.7%, respectively; 16 h later, over 50% of the adult worms isolated from liver tissue showed exposure of worm surface antigen [19, 54, 55]. These results suggest that the susceptibility of different developmental stages of *S. japonicum* to praziquantel is correlated with the exposure of worm surface antigen.

### Uptake of [^3^H]praziquantel by different developmental stages of schistosomes

It has been shown that when adult schistosome-infected mice are treated orally with [^3^H]praziquantel, the radioactivity levels of the worms reach a maximum 0.5–1 h after administration, and then decline markedly 4 h later. In vitro, the uptake of [^3^H]praziquantel by bisexual worms has also been shown to be prompt and increase with the concentration of the drug. After these worms were transferred to a drug-free medium for 15 min, radioactivity decreased by 71–80%, indicating that [^3^H]praziquantel enters into the worms by a simple diffusion mechanism. This is also confirmed by determining the amount of praziquantel using high performance liquid chromatography [56–58].

In a further study, mice infected with various stages of schistosomes were treated orally with [^3^H]praziquantel. Thirty minutes to four hours after treatment, the amount of the silver particles detected in tissues of day 0 worms was significantly lower than the corresponding groups of schistosomes of other stages. This suggests that the susceptibility of different developmental stages of schistosomes to praziquantel is not necessarily related to the amount of praziquantel taken by the worms [59].

## Praziquantel-induced muscular and tegumental effects on schistosomes are Ca^2+^ dependent

### Ca^2+^ influx induced by praziquantel

In vitro studies have demonstrated that spasmodic contraction of *S. mansoni* and *S. japonicum* worms depends on the existence of Ca^2+^, and is inhibited by high concentrations of extracellular Mg^2+^. The rapid contractile activity of male *S. mansoni* worms induced by praziquantel has been explained by the change in the permeability of the parasite to Ca^2+^, which results in an increased calcium influx into the worm and an induction of sustained contraction of worm musculatures. Praziquantel also stimulates the influx of Na^+^, but decreases the influx of potassium ion (K^+^) [20, 24, 28, 29, 60].

It has been shown that when adult *S. mansoni* worms maintained in Hanks’ balanced salt solution (HBSS) with ^45^Ca^2+^ for 40 min were transferred to a zero Ca^2+^ HBSS, a biphasic efflux of ^45^Ca^2+^ from the parasites was observed, i.e., rapid decrease in the first 2 min, followed by a slower rate in the next 40 min. The amounts of total calcium in worms incubated in HBSS and calcium-free HBSS for 1 h were 3.9 ± 0.5 to 2.7 ± 0.4 mmol/kg wet weight, respectively, indicating that about 30% of a worm’s calcium is exchangeable. This exchangeable fraction may play an important role in maintaining the contractile activity of the schistosome [61, 62].

Initially, it has been suggested that vesicle formation induced by praziquantel is independent of the concentration of the external calcium, but subsequent studies have demonstrated that vesicle formation is dependent on the presence of calcium in the external medium [63]. Further studies have shown that when male *S. japonicum* worms were maintained at 37 °C in HBSS with 1.4 mmol/L of ^45^Ca^2+^ for 30 min before praziquantel was added, or both the drug and ^45^Ca^2+^ were added to the medium simultaneously, the ^45^Ca^2+^ uptake of the worms increased significantly within 1–30 min. Thereafter, accumulation of ^45^Ca^2+^ in the worms was no longer observed [64]. On the other hand, if worms were exposed to praziquantel at 4 °C for 2 h, a sustained increase in ^45^Ca^2+^ content in the worms was observed, but no tegument damage or spasmodic contraction was detected. When worms maintained at 4 °C during exposure to praziquantel were transferred to 37 °C, a strong spasmodic contraction occurred and numerous vesicles appeared on the tegumental surface within 15–30 min. Thus, temperature appears to be an important factor in inducing contractile activity or the development of vesicles [64, 65].

Further studies have shown that at 37 °C, the percentage of ^45^Ca^2+^ distributed in the tegumental cytoplasm decreased, while that in the musculature increased, but no change was detected in the total calcium content of a worm. No such phenomenon was observed at 4 °C. Thus movement of calcium between parts of the worm is what may cause praziquantel-induced contraction and tegumental damage, rather than an influx of calcium from the medium [66].

### Voltage-gated Ca^2+^ channels (VGCCs)

Although praziquantel clearly affects Ca^2+^ homeostasis in worms, the exact action mechanism is still not known. Kohn et al. [67] have suggested that VGCCs, e.g., heteromultimeric membrane protein complexes consisting of a pore-forming, voltage-sensing α_1_ subunit, may regulate intracellular Ca^2+^ levels and represent a possible site of action for the drug.

Three high voltage-activated Ca^2+^ channel α_1_ subunit cDNAs were cloned from *S. mansoni.* One of these sequences most closely resembles the L-type class of high voltage-activated α_1_ subunits. The other two sequences are most closely related to non L-type α_1_ subunits. The other two Ca^2+^ channel β subunits were also cloned and expressed, one from *S. mansoni* and the other from *S. japonicum.* These two β subunits (*Sm*Ca_v_βA and *Sj*Ca_v_β) have structural motifs that differ from those found in other known β subunits, and co-expression of these with a mammalian α_1_ subunit conferred sensitivity of the latter to praziquantel. The primary site of β subunit interaction with α_1_ subunits is the β interaction domain (BID). The β BIDs of *Sm*Ca_v_βA and *Sj*Ca_v_β lack two conserved serines that each constitute a consensus site for protein kinase C phosphorylation, and the absence of these serines appears to render schistosome cells sensitive to praziquantel [67–70].

Although several questions remain to be addressed, the hypothesis dealing with the importance of VGCCs as a possible molecular target of praziquantel against schistosomes has been proposed [71]. Voltage-operated Ca^2+^ channels (VOCCs) mediate an extracellular Ca^2+^ influx in muscle fibers of *S. mansoni* worms and, along with Ca^2+^ mobilization of sarcoplasmic reticulum, contribute to muscle contraction [72]. On the other hand, it is known that schistosomes cannot synthesize purine nucleosides de novo, however, in vitro praziquantel may inhibit nucleoside uptake by schistosomes but not by mammalian cells. Adenosine is known to bind to specific receptors and to behave as an indirect antagonist of calcium release in mammalian cells. If calcium channels are correlated with adenosine receptors also in schistosomes, this would support the hypothesis that praziquantel-induced calcium influx may be correlated with an adenosine receptor blockade [73].

There are two contrasting views on whether schistosome VGCCs are involved in the mechanism of action of praziquantel. Valle et al. [74] have indicated that the sequences of cDNAs coding for the *Sm*Cavβ1 and *Sm*Cavβ2 subunits of different sensitive and resistant strains have been cloned and expressed, but no meaningful differences were detected. They were also unable to demonstrate major quantitative differences in the expression of the β subunits obtained from various strains and various developmental stages of *S. mansoni* worms [74]. In another report, the authors used a unique and indirect way to support this hypothesis. They developed an assay based on the transcriptional response of *S. mansoni* worms to heat shock to confirm that day 42 schistosomes in mice are sensitive to praziquantel, which is not the case for day 28 schistosomes. Meanwhile, this sensitivity develops for day 37–day 40 schistosomes, suggesting that the differential effects of praziquantel on day 28 and day 42 worms are not based on cell exclusion as praziquantel can enter the cells of these two stages of schistosomes [75]. Meanwhile, a species of free-living flatworms (*Dugesia japonica*) was used to test the hypothesis of the action mechanism of praziquantel. The result provides the first genetic evidence implicating a molecular target crucial for in vivo praziquantel activity and supports the VOCC hypothesis of praziquantel efficacy [76].

In order to test the hypothesis that calcium channels of schistosomes are the targets for the action of praziquantel, adult *S. mansoni* worms were pre-exposed to the calcium channel blockers nicardipine and nifedipine for 1 h in vitro, followed by adding a praziquantel dose of 3 μmol/L, which is supposed to kill the majority of the schistosomes, and continuous incubation overnight. The worms were then washed and transferred to a drug-free medium for observation for the following 7–10 days. About 50% of schistosomes survived the praziquantel exposure. Further pre-exposure of schistosomes to the actin depolymerizing agent, cytochalasin D, resulted in the parasites being completely refractory to the effects of very high concentrations of praziquantel of up to 36 μmol/L. Similar results were also obtained using adult *S. japonicum* worms. Meanwhile, examination using SEM showed that there was no or slight damage to the tegument surface and gynecophoral canal of the worms pre-exposed to cytochalasin D or nicardipine and nifedipine, which survived in the critical concentration of praziquantel. All these facts are consistent with the hypothesis that schistosome calcium channels may be involved in the action mechanism of praziquantel [77–79].

In a subsequent study, however, Pica-Mattoccia et al. [80] found that exposure of schistosomes to praziquantel after pre-incubation with cytochalasin D not only allows the complete survival of the parasites, but is accompanied by an even higher calcium uptake. Nicardipine and nifedipine also failed to prevent the calcium influx induced by praziquantel. These results place some doubt on the crucial role of Ca^2+^ influx in the antischistosomal activity of praziquantel and on the importance of VGCCs, speculated as the possible molecular target of praziquantel against schistosomes [12, 80]. In recent years, nifedipine has been found to be effective against adult and juvenile *S. mansoni* worms in vitro, which is different from praziquantel. Hence, the authors support the idea of Ca^2+^ subunit as drug targets, but so far no in vivo data are available [81].

### Relationship between efficacy and plasma concentration of praziquantel

It is generally believed that the toxicity and efficacy of drugs are often closely related to their concentration in the host’s blood. Praziquantel is best absorbed from the duodenum and ileum, relatively well from the rectum, and much less so from the colon or stomach. When praziquantel has been injected into various segments of alimentary canal of rabbits infected with *S. japonicum*, the hepatic shift of schistosomes was best from duodenal administration and worst from colon and stomach injection [82].

Adult *S. japonicum* worms lodge in the vessels of the portal vein system, but the relationship between blood concentration and efficacy of praziquantel is unclear (see Table 3). In both mice and rabbits infected with schistosomes for 4 weeks and treated orally with praziquantel at a single dose of 300 mg/kg (mice) or 40 mg/kg (rabbits), the worm reduction rates were similar; in mice the peak concentration of praziquantel in peripheral plasma was 20.2 μg/ml and in rabbits it was only 0.05 μg/ml. When praziquantel (40 mg/kg) was injected into the duodenum of rabbits infected with schistosomes for four or 8 weeks, higher praziquantel concentrations of 16–19 μg/ml were detected in the plasma of the portal vein 15 min after administration. In the subsequent 0.5–8 h, drug concentrations declined from 9 to 15 μg/ml to 0.6–1.3 μg/ml, while those in the femoral vein 5–15 min after administration were as low as 0–0.22 μg/ml and 0.13–0.56 μg/ml [83]. The results demonstrate that praziquantel is extensively metabolized in the first pass through the liver. When aforementioned schistosome-infected rabbits were intramuscularly injected with praziquantel at a dose of 20 mg/kg, the efficacy was similar to when the drug was given orally at a single dose of 40 mg/kg. Interestingly, 5 min to 4 h after intramuscular administration, the plasma concentrations of praziquantel in portal vein blood and femoral vein blood were 1.2–2 μg/ml and 0.6–2.9 μg/ml, respectively, and then declined to 0.6–0.9 μg/ml 8 h later [84]. It was found that in the worm pairs exposed to low concentrations of praziquantel (0.1–1 μg/ml) for 8 h and which were then transferred to a drug-free medium for continuous incubation, their motor activity recovered to norma1 24 h later. If the worm pairs were exposed to praziquantel at a higher concentration of 10 μg/ml for various intervals within 24 h, the worms could not recover their normal activity, resulting in male and female worms dying 6–7 days after incubation.

**Table 3:** Pharmacokinetics and therapeutic efficacy of praziquantel in *S. japonicum*-infected mice and rabbits after oral (PO), rectal (PR) and intramuscular (IM) administration

Animal	Time after infection (week)	Dose (mg/kg)	Route	Tmax (h)	Cmax (μg/ml)	T_1/2_ (h)	AUC_0-∞_ (μg/ml × h)	Worm burden reduction (%)	Reference
Mice	4	300	PO	0.27	20.2	0.8	30.6	46.2	
	5	300	PO	0.59	29.1	0.5	49.8	65.3	
	5	150	IM	0.58	31.3	0.7	60.3	52.2	
	6	300	PO	0.50	34.0	0.6	55.2	93.8	
Rabbits	4	40	PO	0.3	0.05	0.9	0.08	60.8	
	8	40	PO	0.34	0.64	3.04	3.03	94.9	
	12	40	PO	0.43	0.50	1.08	1.01	89.0	
	4	20	IM	1.49	1.92	1.27	8.17	70.9	
	8	20	IM	1.32	1.65	1.86	7.59	88.0	
	12	20	IM	1.11	1.45	1.35	5.25	95.2	[83]
	8	40	PO	0.5–0.6	0.1–0.3	1.3–1.6	0.1–0.9	72.5	
	8	40	PR	0.4	0.7–0.8	0.5–0.6	0.9–1.0	90.1	
	8	20	IM	0.5–1.2	0.8–2.3	1.2–1.6	2.6–5.9	92.2	[84]

The same is true in in vivo studies, e.g., in bisexual schistosomes collected from *S. japonicum*-infected mice treated orally with praziquantel at a lower single dose of 100 mg/kg at various intervals within 72 h and incubated in a drug-free medium for 3 days, most of the worms could recover to normal activity. If bisexual worms were collected from the infected mice treated with praziquantel at a higher single oral dose of 500 mg/kg for 8–72 h, most of the bisexual worms failed to return to normal activity [25]. In another study, three groups of rabbits infected with *S. japonicum* for 8 weeks were treated with a single dose of praziquantel by oral (40 mg/kg), rectal (40 mg/kg), or intramuscular (20 mg/kg) administration. Although the dosage of praziquantel administered intramuscularly was only half that given orally or rectally, the maximum plasma concentration (Cmax) and the area under the concentration-time course curve (AUC) of praziquantel after intramuscular administration was 5–10-fold higher than after oral or rectal administration. Nevertheless, the therapeutic effect of intramuscular administration was not greater than that achieved by rectal administration. On the other hand, the Cmax and AUC in those that were administered rectally were slightly higher than those administered orally, however, the therapeutic effects of these two groups were similar (see Table 3). Furthermore, after praziquantel was administered to schistosome-infected rabbits via the intraduodenal or intramuscular route, the pattern of the drug distributed in the plasma of portal vein and femoral vein was similar to those mentioned above. Meanwhile if the drug was administered rectally, the tendency of praziquantel distributed in the plasma of portal vein and femoral vein was similar to that shown in those that were administered via the intraduodenal route [84].

All these results indicate that no direct correlation exists between route of administration, or between levels of praziquantel in peripheral or portal venous blood, and therapeutic effect [83, 84]. The results confirm the importance of the time the parasite is exposed to the drug [43, 85].

## Efficacy of praziquantel is dependent on host immune responses

It has been known that many antischistosomal drugs, including praziquantel, are dependent on host immune status and immune effector mechanisms, particularly antibodies. Many experimental studies have been carried out in *S. mansoni*-infected mice immunosuppressed by T-cell deprivation or B-cell depleted mice, and the results have been fully summarized in several reviews [86–89]. Here, we describe only the data dealing with the role of host immune response during exposure of *S. japonicum* to praziquantel either in vivo or in several immune systems in vitro.

### Adult schistosomes

When male schistosomes were maintained in a culture medium containing immune rabbit serum (IRS) and a praziquantel concentration of 1 or 30 μg/ml, a granular flocculent material appeared on the drug-damaged surface of the worms. This flocculent material aggregated to form a membrane-like sheath around the worm that accentuated tegumental injury. If IRS was replaced by normal rabbit serum (NRS), no such phenomenon was observed. In terms of male worms exposed to a praziquantel dose of 1 μg/ml for 4 h and then transferred to a drug-free medium containing IRS, most schistosomes recovered. When male worms were exposed to praziquantel at a higher concentration of 30 μg/ml for one or 4 h before being transferred to a medium containing NRS, the damaged tegument was repaired and worm activity returned to normal in one half of the worms tested. When IRS replaced NRS, most of the worms did not recover. When male, perfused out from infected mice 1–8 h after treatment with praziquantel (single oral dose of 50 mg/kg) were transferred to a medium containing NRS or IRS and incubated for another 3 days, all or most of the damaged worms recovered to normal. In the worms obtained from infected mice treated with praziquantel at a higher dose of 400 mg/kg for 4–8 h and then transferred to a medium containing IRS, neither apparent recovery of tegumental damage nor worm activity were detected. Regarding the worms transferred to the medium containing NRS, some of the worms recovered, at different degrees [90].

Furthermore, for bisexual schistosomes maintained in a medium containing IRS or rabbit antisera to frozen-thawed tegument exudates of adult worms (ASE), glycogen-activated mouse peritoneal neutrophils and complement were exposed to a praziquantel dose of 1 μg/ml for 2–20 h, neutrophils, mediated by the aforementioned membrane-like sheath were found attached to the worm’s surface. If male and female worms were first exposed to a praziquantel concentration of 30 μg/ml for 1–4 h, then placed in the above medium without the drug, neutrophils were also observed to attach to the damaged tegumental surface of the worms. Attachment of the neutrophils appeared to accentuate tegumental damage, which resulted in worm death within 24 h. No such phenomenon was observed when the immune serum was replaced by NRS [91]. Furthermore, if a Boyden chamber was used, an increase in neutrophil chemotactic activity, induced by praziquantel-damaged worms, was detected [92]. Half an hour after administering a praziquantel dose of 300 mg/kg to infected mice, the tegument of schistosomes showed swelling and vacuolization at various degrees, followed by a rupture of vacuoles and formation of flocculus materials that attached to the damaged surface of the worms, similar to that seen in schistosomes maintained in the medium containing immune serum and praziquantel. Six hours after medication, numerous polymorphonuclear leukocytes attached to the damaged tegument of the worms and penetrated into the worm’s body 12 h later. Meanwhile, the appearance of pathological changes of the worm tegument caused by praziquantel was seen earlier in male worms than in female worms. Nevertheless, 12–16 h after medication, female worms also had severe damage on the tegument, especially on the surface along the ovary and vitelline glands, which is similar to that observed in in vitro studies. Twenty-four hours after medication, male and female worms died [91, 93]. Similar results were obtained for *S. mansoni*-infected mice treated with praziquantel [43].

These findings indicate that antibodies might play an important role in praziquantel-mediated tegumental damage. In vitro, schistosomes exposed to a praziquantel dose of 10 μg/ml might survive for 1–3 days, and the survival time of female worms is even longer than that of males. While in in vivo studies the elimination of praziquantel from the host and worms has been shown to be rapid [23, 25, 57, 94], tegumental damage of the worms persists after praziquantel treatment, indicating that host factor could sustain praziquantel-induced damage in vivo. As a result of exposure of worm surface antigens, *S. japonicum* seems to be more susceptible to a host-mediated immune attack [23, 54].

Various experimental studies have confirmed that the immune level of the host impacts the efficacy of praziquantel. For example, a single dose of 40 mg/kg administered to rabbits infected with 3-, 4-, 8-, or 10-week-old *S. japonicum* resulted in worm burden reductions of 14%, 46%, 84%, and 88%, respectively [95]. Further studies have shown that in rabbits infected with 50, 200, and 400 *S. japonicum* cercariae for four and 8 weeks, and receiving the same single oral praziquantel dose of 40 mg/kg, the worm reduction rates of the 8-week groups were 88%, 92%, and 97%, respectively. Similar results were obtained for mice infected with *S. japonicum* and the antibody level was shown to be positively correlated with duration and intensity of the infection [95, 96].

### Day 0 worms

In a mouse model, a single oral praziquantel dose of 400 mg/kg or 600 mg/kg has a noticeable effect on day 0 schistosomula (skin stage), but has less of an effect or is even ineffective on 6–48-h-old (skin stage) and day 3 (lung stage) schistosomula. In vitro, the same concentration of praziquantel induced similar stimulation of motor activity and spasmodic contraction in day 0, day 1 (24-h-old), and day 3 schistosomula, while the drug-induced tegument damage in day 0 schistosomula was more severe relative to that of day 1 and day 3 schistosomula. Forty-eight hours after infection, a considerable part of day 1 worms still remained in the skin, and a lower susceptibility to praziquantel revealed their intrinsic resistance to praziquantel. Observation using the IFAT showed that the percentages of exposure of surface antigen in day 0, day 1, and day 3 schistosomula were 86.4%, 55.2%, and 3.9%, respectively. It is suggested that the differences in susceptibility of these early stages of *S. japonicum* to praziquantel may be related to antigenic composition of their respective tegumental surfaces. Furthermore, when these three different aged schistosomula were injected into the peritoneal cavity of a mouse containing neutrophils or macrophages, the worm surface of day 0 worms rapidly attached by either kind of cells, but such phenomenon was not seen in day 1 and day 3 schistosomula. Similar results were also observed in in vitro studies, indicating differences in body surface properties at different worm ages [97, 98].

Furthermore, histological observations showed that after treatment with praziquantel, day 0 schistosomula in the host’s skin were infiltrated by inflammatory cells and some of the cells attached to or penetrated into the worm’s body; meanwhile, an increase in degranulation and collapse of the mast cells around the worms occurred. This means that host nonspecific immunity may participate in the killing mechanism during treatment, which is confirmed by the fact that praziquantel can increase the nonspecific immunity of mice after their early-stage infection with cercariae [99].

### Synergistic effects of schistosome antigen in combination with praziquantel

In the 1990s, a *S. japonicum* vaccine combined with praziquantel was used to enhance the killing effect against juvenile and adult schistosomes. Initially, mice were treated orally with praziquantel at a lower dose of 20 mg/kg in combination with intraperitoneal injection of class IgG_1_ monoclonal antibodies ISj51 or ISj55 2 h before challenge with cercariae. The same treatment regimen was repeated 4 days later. Using this combined treatment, the worm reductions were 45.3% and 43%, respectively, which were higher or significantly higher than that of each monoclonal antibody or praziquantel alone [100]. Subsequently, mice received an intraperitoneal injection of monoclonal antibody, McAb14 or McAb24, combined with an oral administration of a praziquantel dose of either 20 or 50 mg/kg 2 h before infection with cercariae; this resulted in worm reductions of 45.4% and 44.2%, respectively, significantly higher than that of 13.9%, 31.5%, or 29.8% obtained for the groups of mice treated with McAb14, McAb24, or praziquantel alone.

Further studies have shown that in mice or rabbits treated with monoclonal antibody SSj14 combined with a low dosage of praziquantel, or SSj14 together with praziquantel and fenofibrate administered 2 h before challenge with cercariae, synergistic effects were observed [101, 102]. Furthermore, when mice were given an intraperitoneal injection of monoclonal antibody against schistosomula membrane 1 day before challenge with cercariae, followed by an intraperitoneal injection with a praziquantel dose of 200 mg/kg on day 3 or day 38 after infection, the worm reduction rates were 91.9% and 96.9%, significantly higher than that of 17.2% and 84.9% as obtained from the corresponding group treated with praziquantel alone. This demonstrates the synergistic effect of praziquantel combined with schistosome monoclonal antibody against juvenile and adult schistosomes [103].

In another experiment, mice infected with *S. japonicum* cercariae for 2 days, 14 days, or 35 days were treated orally with praziquantel at a single dose of 150 mg/kg, followed by intravenous injection of IRS against surface membrane antigen of adult *S. japonicum* via tail vein 30 min after administration of the drug. The results showed that the immune serum increased the antischistosomal activity of praziquantel against day 2, day 14, and day 35 worms by 39%, 30%, and 47%, respectively. In addition, praziquantel in combination with another two monoclonal antibodies, 3B6 and IC2, also exhibited synergistic effects against adult schistosomes. All these data demonstrate that humoral immunity plays an important role in the schistosomicidal process of praziquantel [104, 105].

Meanwhile, the immunological response, such as the level of lymphocyte response to phytohemagglutinin (PHA) and adult worm antigen, the T-cell helper activity as well as IgM released from the antibody forming cell, in mice infected with *S. japonicum* increased after treatment with praziquantel. On the other hand, the immunosuppressive effects of infected hosts were improved after treatment, which suggests that besides the host immune status and antischistosome antibody, cellular immunity may also be involved and modulated in the lethal mechanism of praziquantel on the schistosomes [106]. It means that the death of schistosomes induced by praziquantel is closely related to the role displayed by the host.

## Prevention and early treatment

Chemoprophylaxis can be roughly defined as protecting the definitive host from the penetration of cercariae into the skin. It is known that *S. japonicum* cercariae are more susceptible to praziquantel in water than in isotonic saline. The MEC of praziquantel required to kill cercariae in water is 0.05 μg/ml. Exposure of cercariae to praziquantel results in immediate contraction, followed by an increase in motor activity, release of gland contents, and separation of the tail from the body. Subsequently, the surface of the cercariae is damaged, as indicated by an increased permeability, followed by swelling of the cercarial body. Examination using TEM has shown that the glycocalyx in the outer surface of tegument decreases markedly or even disappears, and hence the cercariae cannot stand the nonisotonic environment water and dies within 2–4 h [107–109]. When cercariae were placed on the skin of mice treated orally with praziquantel 2–4 h prior, most of the cercariae were killed rapidly on the surface or keratin layer of the skin [107, 110]. Meanwhile, the cercariae that penetrated into the dermis or hypodermis showed severe damage and were surrounded by inflammatory cells. Further analysis demonstrated that upon administration of tritiated praziquantel to mice, there was no radioactivity in the skin in the following 2–4 h after; this supported previous findings regarding the effect of praziquantel on cercariae. Eight to 24 h after medication, the radioactivity excreted from the skin revealed no apparent effect against the parasites. Therefore, praziquantel exhibited a prophylactic effect, e.g., oral administration of praziquantel at a single dose of 400 mg/kg given to mice 2–4 h before infection with *S. japonicum* cercariae resulted in worm reduction rates of 88–100%. If the same oral dose of praziquantel was administered to mice 2–4 h after infection, lower worm reduction rates of 36–84% were observed [17, 107]. Nevertheless, when a praziquantel dose of 50 mg/kg was administered intramuscularly to dogs 4 h before and after infection, the worm reduction rates were 61% and 44%, respectively. On the other hand, when praziquantel was given at a higher oral or intramuscular dose to rabbits 4 h before or after challenge with cercariae, poor prophylactic effects were observed [17]. Hence, the window of praziquantel sensitivity for prophylaxis is very narrow, and only confined to mice. Therefore, praziquantel does not have practical significance for prevention of schistosomiasis.

Although praziquantel given at a single curative dose to mice harboring day 21 schistosomula exhibits less efficacy as evaluated by the worm burden reduction, female worms show apparent shrinkage in size, depigmentation of the gut, degeneration of the vitelline glands, atrophy of the ovary, and disappearance of eggs in the uterus. Meanwhile, the oviposition of female worms is inhibited significantly or even ceased during the following 2–3 weeks [111]. Therefore, it is suggested if praziquantel is given once at appropriate intervals several times to the host starting early after infection, the majority or even all the female worms are expected to be killed just after the worms reach maturity and commence egg production. Among the regimens tested, the most promising one showed that when praziquantel was administered 21 days after infection, followed by repeated dosing at 1–3-week intervals 2–3 times, all mice were free from female worms (especially in the 3-week interval group) [111]. In rabbits infected with cercariae once every week six times or once every other day five times, initial treatment with praziquantel on day 21 after the first infection at a single dose of 40 mg/kg followed by administration of the same dose at a two-week interval four or two times resulted in promising effects, with female worm reduction rates of 99–100%. Meanwhile, the presence of adult schistosomes in the same host not only increased the effect of praziquantel against day 21 schistosomula, but also had an effect on day 14 schistosomula [19, 112]. When rabbits were singly infected with cercariae and received the same dose of praziquantel 21 days after infection, followed by repeated dosing at 1–2-week intervals twice, similar results were observed. Meanwhile, the livers of rabbits showed normal or mild changes, and the parameters relevant to acute schistosomiasis were negative as compared to the controls [112]. In rabbits infected with schistosome cercariae once every other day five times, administration of a praziquantel dose of 30 mg/kg was started 21 days after the first infection, followed by repeated dosing at a two-week interval twice. Histopathological examination showed that the reduction in the number of hepatic granulomas were 76.5–85.5%. Meanwhile, the structure of the hepatic lobules was normal [113], indicating that either the host was protected from the infection or there was a great decrease in the intensity of the infection.

## Resistance to praziquantel

Currently, praziquantel is the only globally available antischistosomal, and heavy reliance on a single drug for schistosomiasis control may promote the spread of drug-resistant parasites. Experiments with mice have revealed the possibility of selecting praziquantel-tolerant strains of *S. mansoni* after repeated administration of subcurative doses of praziquantel [114].

A series of laboratory studies and clinical trials conducted in Egypt and Senegal between 1995 and 2002 have raised considerable concern about the possible tolerance or development of resistance to praziquantel in schistosomes [115–120]. Indeed, *S. mansoni* isolates with a somewhat reduced praziquantel susceptibility have been identified by several authors [121–123]. However, these observations are fortunately of limited clinical significance thus far [12, 124].

It has been speculated that antimicrobial resistance is responsible for the unusually low cure rates in *S. mansoni-*infected patients from Senegal [119]. In Egypt, patients were not completely cured of *S. mansoni* infections even after praziquantel was administered three times, which is the most compelling evidence of praziquantel resistance to date [117, 118, 121].

In China, treatment of schistosomiasis japonica with praziquantel began in 1978. In the early 1990s, experimental studies indicated that in infected mice treated repeatedly with praziquantel at a curative dose, or in mice infected with 14–18-day-old schistosomula or adult worms and treated with praziquantel at various dose schedules, followed by the passage in the intermediate host snails, i.e., *Oncomelania hupensis* with miracidia hatching from schistosome eggs laid by the residual female worms, the F_1_ and F_2_ progeny of worms were still susceptible to praziquantel [125, 126]. In recent years, laboratory studies have successfully developed two isolates of *S. japonicum* (mainland China) resistant to praziquantel through continuous treatment with subcurative doses of praziquantel and a total of eight passages in *O. hupensis* snails. Meanwhile, not only are the sensitivities of the adult worms of the two isolates significantly lower than those of the isolates never treated with praziquantel, but cercariae and miracidia are also resistant to the drug [127, 128]. In order to understand and compare the sensitivities of different isolates of *S. japonicum* to praziquantel, *S. japonicum-*shedding snails from marshland and lake regions of Hunan, Hubei, Jiangxi, Anhui, and Jiangsu provinces were used to infect animals with various isolate(s) of cercariae released from the snails in order to determine the ED50 against adult schistosomes 5 weeks later. The results showed that the sensitivities of various isolates of *S. japonicum* to praziquantel are similar without significant differences [129].

From the 1990s to prior to the 2010s, a series of field studies were carried out in endemic villages distributed in six provinces of China, where praziquantel had been used extensively for 10–14 years. The different prevalence rates were found for schistosomiasis: from low (3.46%) to high (8–12%). The results showed that *S. japonicum* is still highly susceptible to praziquantel. Meanwhile, when praziquantel was administered to patients at a single oral dose of 40 mg/kg, the efficacy of praziquantel in the area with repeated chemotherapy was not significantly different from that in the newly identified endemic foci [130–137].

All these results suggest that in large areas of China, after extensive and repeated use of praziquantel in the treatment of *S. japonicum*-infected individuals or mass chemotherapy given to persons in highly endemic areas without preliminary screening for about three decades among tens of million person-times in a huge population, no evidence of tolerance or resistance of *S. japonicum* to praziquantel has been found.

Interestingly, when the area in Egypt at which resistant isolates were identified was revisited to examine the current efficacy of the drug using the same treatment protocol after 10 years of therapeutic pressure, sustained efficacy was recorded despite the presence of schistosome isolates with diminished sensitivity to the drug [138].

## Conclusions

In 1918, PAT initiated the era of chemotherapy for schistosomiasis. Various categories of antischistosomal chemicals have been synthesized and developed since then. However, only a few of these have been introduced for clinical trials. After clinical practice for four decades globally, praziquantel has been confirmed as a very effective and almost the only drug of choice for the treatment of human schistosomiasis. Indeed, the discovery of praziquantel is a milestone, and extensive use of the drug has made a great contribution to schistosomiasis control worldwide.

Experimental studies have shown that in vivo effective antischistosomal drugs, such as PAT, amoscante, oxamniquine, niridazole, furapromidum, mefloquine, and artemisinins can cause a hepatic shift and tegumental damage of schistosomes within 1–3 days post oral administration. However, only praziquantel has a similar effect against schistosomes either in vitro or in vivo at a very low concentration and a very rapid onset. Particularly, the damage to the tegument creates the condition and basis for the host to start the process of killing the worms. Therefore, the effects of praziquantel against schistosomes should have some reference significance for the study of new antischistosomal drugs.

Oral administration of praziquantel to *S. japonicum*-infected animals exhibits no apparent correlation between therapeutic efficacy and drug concentration in peripheral venous blood, but the relationship between the concentration of the drug in the blood of mesenteric vein and portal vein and therapeutic efficacy is more important [83, 84]. Praziquantel is absorbed mainly from the small intestine [82] and thereafter schistosomes are exposed to it, lose their power to lodge in the superior mesenteric vein and portal vein, and shift back to the liver. As the drug possesses a strong first pass effect in the liver where > 90% of the drug is metabolized (supported by the low but satisfactory drug level in the peripheral circulation and high blood level in the portal vein), worms distributed in inferior mesenteric veins and its branches are insulted and quickly shift to the liver.

The pharmacokinetics property of furapromidum [3, 139, 140] is similar to praziquantel. Unfortunately, the very low drug concentration in peripheral blood is not enough to drive the schistosomes distributing in inferior mesenteric veins and its branches to shift to the liver, which results in a lower cure rate obtained by treatment with furapromidum both in rabbits and humans with *S. japonicum* infection [140–142].

The exact action mechanism of praziquantel is still not fully known. Although scientists have made efforts to understand the action of praziquantel on the VGCCs of *S. mansoni*, opinions remain inconsistent. Praziquantel is less effective in T-cell deprived mice or B-cell depleted mice [86–89]. Meanwhile, a very strong first pass effect of praziquantel through the liver, accompanied by a half-life of a few hours in various animals [143] or 1.5 h in humans [144] is unfavorable for treatment. But exposure of the worm surface antigen induced by praziquantel triggers the host action of immune effector mechanisms, which plays a synergistic role and results in the death of the worms. Hence, the action mechanism of praziquantel against schistosomes should include the target positions or molecules attacked by the drug, and immune reaction derived from the host. It is thus necessary to conduct in-depth studies on the host action of immune effector mechanisms in the process of killing schistosomes by praziquantel.

Praziquantel was selected from over 400 1,2,3,6,7,11b-hexahydro-4H-pyrazino [2,1-α] isoquinolin-4-one and related compounds because of its outstanding anthelmintic properties [143]. The chemical structure of praziquantel has been extensively modified by many scientists, but they have failed to develop a better alternative compound, which has been fully summarized in a recent review [144]. So far, many categories of synthesized compounds have been reported to be effective against schistosomes [7, 145–147]; a few are related to each other, while most are different types. However, the same types of compounds often exhibit very high structural specificities, i.e., modification of an effective compound always obtains the compounds with little effect over the original one. Hence, it is still necessary to design a new category of compounds for the development of new antischistosomal drugs.

## Additional file

Additional file 1: Multilingual abstracts in the five official working languages of the United Nations. (PDF 783 kb)

## Abbreviations

AKP: Alkaline phosphatase
AUC: Area under concentration-time course curve
BID: β interaction domain
Ca^2+^: Calcium ion
Cmax: Maximum plasma concentration
HBSS: Hanks’ balanced salt solution
IFAT: Indirect fluorescent antibody technique
IRS: Immune rabbit serum
K^+^: Potassium ion
LM: Light microscopy
MEC: Minimal effective concentration
Mg^2+^: Magnesium ion
Na^+^: Sodium ion
NRS: Normal rabbit serum
OZ: Ozonide
PAT: Potassium antimony tartrate
RMP: Resting membrane potential
SEM: Scanning electron microscopy
TEM: Transmission electron microscopy
VGCC: Voltage-gated Ca^2+^ channel
VOCC: Voltage-operated Ca^2+^ channel
