# Supplementary material for: Pharmacological and immunological effects of praziquantel against Schistosoma japonicum: a scoping review of experimental studies
Source: Infect Dis Poverty. 2018 Feb 7;7:9. doi: 10.1186/s40249-018-0391-x (PMC5801800; doi:10.1186/s40249-018-0391-x)
Supplement: Additional file 1: — Multilingual abstracts in the five official working languages of the United Nations. (PDF 783 kb) [file 40249_2018_391_MOESM1_ESM.pdf]

Translation of the abstract into the five official working languages of the United Nations

الآثار الدوائية والمناعية للبرازيكوانتيل ضد البلهارسيا اليابانية: استعراض لتحديد النطاق من الدراسات التجريبية

شو-هوا، وزياو، وجون صن، ومينغ-غانغ تشن

الملخص
خلفية: يتوفر العلاج الكيميائي لمرض البلهارسيا منذ حوالي 100 سنة. خلال القرن الماضي، بُذلت جهود كبيرة لتطوير عقاقير جديدة مضادة للبلهاريسيا بدءً من الأنتيمونيات إلى اللاأنتيمونيات، وقد استخدم بعضها على نطاق واسع في العلاج السريري. وباستثناء عدد قليل من الأدوية، مثل أوكسامنيكين والميتريفونات، فإن معظم مضادات البلهارسيا التي ظهرت في فترة ما قبل البرازيكوانتيل كان لها قيود متغيرة فيما يتعلق بالسلامة والفعالية. وبرغم استخدام الأوكسامنيكين والمتريفونات لمكافحة البلهارسيا، إلا أنها فعالة فقط ضد البلهارسيا اليابانية والبلهارسيا المنسونية و البلهارسيا الدموية، على التوالي. حاليا، أصبح البرازيكوانتيل هو الدواء الوحيد المستخدم لعلاج جميع أنواع البلهارسيا البشرية الخمسة. في هذا الاستعراض، لخصت ونوقشت الآثار الدوائية والمناعية للبرازيكوانتيل ضد البلهارسيا اليابانية .
النص الأصلي: منذ نهاية السبعينات وحتى العقد الأول من القرن العشرين، أجرى العلماء سلسلة من الدراسات التجريبية على تأثيرات البرازيكوانتيل ضد البلهارسيا اليابانية. وقد تضمنت هذه الدراسات فحص وظيفته الدوائية الفريدة على البلهارسيا، والخصائص في استعداد المراحل النمائية المختلفة للبلهارسيا تجاه الدواء، والعلاقة بين تركيز البلازما للدواء وفاعليته، وتأثير العوامل المضيفة على عمل الدواء في إبادة البلهارسيا، والوقاية والعلاج المبكر لعدوى البلهارسيا، فضلا عن البلهارسيا المقاومة للدواء.
الاستنتاج: قد تكون آثار البرازيكوانتيل ضد البلهارسيا اليابانيةكما أوضحت الدراسات التجريبية التي استعرضت في هذه الورقة، مشتملة على بعض الأهمية المرجعية لتطوير مضادات البلهارسيا الجديدة.

Translated from English version into Arabic by Zahraa_Hesham, proofread by Mahmoud Sami, through

TRANSLATORS
WITHOUT BORDERS

吡喹酮抗日本血吸虫的药理和免疫效应的实验研究综述

肖树华，孙军，陈名刚

摘要
引言：血吸虫病的药物治疗已有约 100 年的历史。在过去一百年中，从锑剂到非锑剂，抗血吸虫药物有了很大的发展，其中一些药物曾广泛用于临床治疗。但除少数几种药物外，如奥沙尼喹和敌百虫，其它大部分抗血吸虫药物都存在安全性和有效性方面的诸多问题。尽管奥沙尼喹和敌百虫曾用于血吸虫病防治，但是它们仅对曼氏血吸虫和埃及血吸虫有效。目前吡喹酮是唯一同时对 5 种感染人体的血吸虫有效的药物。
内容：从 1970 年末到 2000 年，中国科学工作者对吡喹酮抗日本血吸虫的作用进行了一系列的实验研究，内容包括吡喹酮的独特抗血吸虫作用、不同发育期血吸虫对吡喹酮敏感性的特征、血药浓度与疗效的关系、宿主因素对吡喹酮杀虫作用的影响、预防血吸虫感染和早期治疗，以及血吸虫对吡喹酮的抗性等。

结论：本文对吡喹酮抗日本血吸虫的作用特点进行了综述和讨论，对发展抗血吸虫新药具有参考意义。

Translated from English version into Chinese by Jun Sun

Effets pharmacologiques et immunologiques du praziquantel contre le *Schistosoma japonicum* : examen de la portée des études expérimentales

Shu-Hua Xiao, Jun Sun, Ming-Gang Chen

Résumé

Contexte: La chimiothérapie contre la schistosomiase existe depuis 100 ans. Au cours du siècle dernier, des efforts considérables ont été engagés pour mettre au point de nouveaux médicaments antischistosomiques, antimoniaux et non-antimoniaux, et certains d'entre eux ont été largement utilisés dans le traitement clinique. À l'exception de quelques médicaments, tels que l'oxamniquine et le métrifonate, la plupart des antischistosomaux développés au cours de la période pré-praziquantel présentent des limitations variables en termes d'innocuité et d'efficacité. Bien que l'oxamniquine et le metrifonate aient été utilisés pour le contrôle de la schistosomiase, ils ne sont efficaces que contre le *Schistosoma mansoni* et le *S. haematobium* respectivement. Actuellement, le praziquantel est le seul médicament utilisé pour traiter les cinq espèces de schistosomes humains. Cet examen de portée résume et explore les effets pharmacologiques et immunologiques du praziquantel contre le *S. japonicum*.

Texte principal: De la fin des années 1970 jusqu'aux années 2000, des scientifiques ont mené une série d'études expérimentales sur les effets du praziquantel contre le *S. japonicum*. Ils ont examiné son action pharmacologique unique sur les schistosomes, les caractéristiques de sensibilité des schistosomes face au médicament aux différents stades de leur développement, la relation entre la concentration plasmatique du médicament et son efficacité, l'impact des facteurs propres à l'hôte sur l'action biocide du médicament, la prévention et le traitement précoce de l'infection schistosomique, ainsi que la schistosomiase résistante au praziquantel.

Conclusion: Les effets du praziquantel contre le *S. japonicum*, tels qu'élucidés par les études expérimentales examinées dans cet article, peuvent servir de point de référence pour le développement de nouveaux antischistosomaux.

Translated from English version into French by charlotteman, proofread by claisney, through

TRANSLATORS
WITHOUT BORDERS

Фармакологическое и иммунологическое воздействие празиквантела на японскую шистосому *(Schistosoma japonicum)*: Аналитический обзор экспериментальных исследований

Шу-Хуа Сяо, Цзюнь Сунь, Мин-Ган Чэнь

Аннотация

Справочная информация: Химиотерапия при шистосомозе применяется уже на протяжении 100 лет. В прошлом столетии были предприняты значительные усилия для разработки новых сурьмяных и иных шистосомоцидных средств, некоторые из которых активно использовались в клинической терапии. За исключением нескольких лекарственных препаратов, таких как оксамнихин и метрифонат, большинство шистосомоцидных средств, приходящихся на период до разработки празиквантела, обладали рядом ограничений как в отношении безопасности, так и по эффективности их применения. Несмотря на то, что оксамнихин и метрифонат до сих пор применяются для борьбы с шистосомозом, оба указанных препарата эффективны исключительно для лечения шистосомы Мансона *(Schistosoma mansoni)* и кровяной шистосомы *(S. haematobium)*соответственно. В настоящее время празиквантел является единственным лекарственным препаратом, применяемым при лечении всех пяти разновидностей человеческой шистосомы. В данной работе обобщается и анализируется фармакологическое и иммунологическое воздействие празиквантела на японскую шистосому *(Schistosoma japonicum)* .

Основная часть: С конца 1970-х годов и до 2000-го года учёными были проведены серии экспериментов, направленных на изучение воздействия празиквантела на японскую шистосому *(S. japonicum)*. В указанные экспериментальные исследования входили: изучение уникального фармакологического воздействия данного препарата на шистосомы, особенности их восприимчивости к этому лекарственному средству на различных стадиях жизненного цикла, взаимосвязи между концентрацией препарата в плазме и его эффективностью, влияния факторов организма носителя на реакцию на лекарство, профилактики и лечения шистосомоза на ранних стадиях, а также шистосомоза, устойчивого к празиквантелу.

Заключение: Как отмечалось в экспериментальных исследованиях, обзор которых произведён в настоящей работе, информация о воздействии празиквантела на японскую шистосому *(S. japonicum)*может нести справочный характер при разработке новых шистосомоцидных препаратов.

Translated from English version into Russian by Liudmila Tomanek, proofread by TatianaKary, through

TRANSLATORS
WITHOUT BORDERS

Efectos farmacológicos e inmunológicos del praziquantel en contra *Schistosoma japonicum*: Una revisión exploratoria de los estudios experimentales

Shu-Hua Xiao, Jun Sun, Ming-Gang Chen

Resumen

Introducción: La quimioterapia en contra de la esquistosomiasis ha estado disponible durante 100 años. Durante el siglo pasado se han hecho grandes esfuerzos para desarrollar nuevos fármacos antiesquistosoma, desde los derivados antimoniales hasta los no antimoniales, y algunos de ellos han sido utilizados extensamente en el tratamiento clínico. Con la excepción de algunos fármacos, como la oxamniquina y el metrifonato, la mayoría de los antiesquistosomales generados antes que el graziquantel, tienen diferentes limitaciones con respecto a su eficacia y seguridad. Aunque la oxamniquina y el metrifonato se han utilizado para el control de la esquistosomiasis, sólo han sido efectivos contra *Schistosoma mansoni* y *S. haematobium*, respectivamente. Actualmente el praziquantel es el único medicamento utilizado para el tratamiento de las cinco especies de esquistosomas en humanos. En esta revisión, se analizan y resumen los efectos farmacológicos e inmunológicos del praziquantel contra *S. japonicum* .

Texto principal: Desde finales de los años 70 hasta los primeros años del siglo XXI, los científicos han realizado una serie de estudios experimentales sobre los efectos del praziquantel contra *S. japonicum*. Estos han incluido: el examen de la acción farmacológica única sobre los esquistosomas, las características de la susceptibilidad en las diferentes etapas del desarrollo en los mismos esquistosomas, la relación entre la concentración plasmática del fármaco y su eficacia, el impacto de los factores ligados al hospedador, a la acción bactericida del fármaco, a la prevención y el tratamiento precoz de la infección esquistosomial, así como la esquistosomiasis resistente al praziquantel.

Conclusión: Los efectos del praziquantel contra *S. japonicum*, tal como se deduce de los estudios experimentales revisados en este trabajo, pueden tener algún marco de referencia significativa para el desarrollo de nuevos antisquistosomales.

Translated from English version into Spanish by jjmoreiras, proofread by mariatacost, through

TRANSLATORS
WITHOUT BORDERS
